# Co-RESPOND - a European federated network of longitudinal cohorts investigating the effects of the Covid-19 pandemic on mental health and resilience

**DOI:** 10.1192/j.eurpsy.2023.2198

**Published:** 2023-07-19

**Authors:** J. Stoffers-Winterling, P. Petri-Romão, C. Doerschner, M. Melchior, M. Sijbrandij, R. Kalisch, K. Lieb

**Affiliations:** 1 Leibniz Institute for Resilience research, Mainz, Germany; 2 Department of Social Epidemiology (ERES), Sorbonne Université - Faculté de Médecine, Paris, France; 3 Vrije Universiteit Amsterdam, Amsterdam, Netherlands

## Abstract

**Introduction:**

European researchers are collaborating in the EU Horizon 2020-funded project “RESPOND” to address the psychological and psychosocial effects of the Covid 19 pandemic in order to prepare health systems for future crises. In the Co-RESPOND subproject, several longitudinal cohorts are contributing to an individual participant data (IPD) meta-analysis.

**Objectives:**

Co-RESPOND aims to assess trajectories of mental health and resilience, and to identify relevant moderators using a meta-analysis of individual participant data (“IPD”) approach. Moreover, a research network of European cohorts is being established alongside a sustainable shared IT infrastructure. Co-RESPOND aims to publish the results of the collaboration in a findable, accessible, interoperable and reusable way according to the “FAIR publication” principles.

**Methods:**

To achieve these aims, a federated network for remote data analysis is being built. In this talk we describe the steps necessary to join existing cohorts into one network, and which challenges need to be met: First, existing data sets need to be harmonized retrospectively, second, data sharing and processing needs to be done in accordance with the GDPR requirements, and third, a technical solution needs to be found to facilitate joint analyses and publication of the network and its products.

**Results:**

We identified the Maelstroem guidelines for retrospective data harmonisation of epidemiologic studies as appropriate guidance to carry out and document the transformation of individual data sets. The OBiBa software suite is used to build the IT infrastructure of the project by connecting local data servers of the study sites and making them available for remote analyses by other partners. As of autumn 2022, data transformation is finalized and data sets uploaded on the local servers. A platform on the internet is created where the main characteristics of all participating cohorts (“meta-data”) are catalogued to help them gain visibility and make them findable for future joint projects. The Co-RESPOND network will be open for more partner cohorts to join.

**Conclusions:**

The Covid pandemic has stimulated lots of international remote collaborations, and federated networks for data analyses are increasingly used as a means of enhancing the value of existing data sets. User-friendly and cost-free software solutions are already available (e.g., OBiBa) to facilitate such endeavours. However, researchers intiating cohort studies should be aware of such technology and methods and consider the use of their data in overarching collaborations from the start. We conclude with concrete recommendations how to optimize the design of epidemiologic data collections to enhance their interoperability with other cohorts, e.g., by using international coding standards.

**Disclosure of Interest:**

None Declared

